# RON Expression Mediates Lipopolysaccharide-Mediated Dendritic Cell Maturation *via* March-I

**DOI:** 10.3389/fcimb.2020.606340

**Published:** 2021-01-18

**Authors:** Lingtong Huang, Xueling Fang, Xuan Zhang, Weifang Wu, Hangping Yao, Qiang Fang

**Affiliations:** ^1^Department of Critical Care Units, The First Affiliated Hospital, Zhejiang University, School of Medicine, Hangzhou, China; ^2^State Key Laboratory for Diagnosis & Treatment of Infectious Diseases, The First Affiliated Hospital, Zhejiang University, School of Medicine, Hangzhou, China; ^3^Department of Infectious Disease, The First Affiliated Hospital, Zhejiang University, School of Medicine, Hangzhou, China

**Keywords:** dendritic cells, Recepteur d’origine nantais, ubiquitination, March-I, lipopolysaccharide

## Abstract

The macrophage stimulating protein (MSP)–Recepteur d’origine nantais (RON) signaling pathway regulates macrophage function. Here, we verified RON receptor expression in bone marrow-derived dendritic cells (BMDCs) by real time-PCR, Western blot, and flow cytometry. Flow cytometry was used to detect the changes in MHC II and CD86 expression following the inhibition of RON in BMDCs and splenic dendritic cells (DCs). Immunoprecipitation and Western blot were used to detect the level of MHC II and CD86 ubiquitination. An enzyme-linked immunosorbent assay was used to detect cytokine release, and a mixed lymphocyte reaction was performed to evaluate DC maturity. The results show that the inhibition of RON leads to an increase in March-1 transcription, which intensifies the ubiquitination of MHC II and CD86 and ultimately leads to a decreased level of these two molecules. The mixed lymphocyte reaction provided evidence that RON inhibition decreased the ability of DCs to promote the proliferation of T cells. The MSP-RON signaling pathway may play an important role in lipopolysaccharide (LPS)-stimulated DC maturation through March-I and may protect DC differentiation following LPS stimulation.

## Introduction

Dendritic cells (DCs) represent a type of antigen-presenting cell that recognizes and eliminates invading pathogenic microorganisms through the activation of Toll-like receptors (TLRs). Following DC stimulation with TLR ligands [*e.g.*, lipopolysaccharide (LPS)], immature DCs turn into mature DCs, exhibited by increased major histocompatibility complex (MHC) II and CD86 expression and enhanced release of inflammatory factors. Mature DCs stimulate the activation of naive T cells. MHC II and CD86 expression in DCs is regulated by ubiquitination and degradation by the E3 ligases March-I and March-8 (Furuta et al., 2013; Oh and Shin, 2015). The expression of March-I in immature DCs enables the cells to recycle MHC II molecules, thereby efficiently treating antigens intracellularly, and loading the antigens onto MHC II. Moreover, MHC II ubiquitination is decreased in LPS-stimulated DCs, which could contribute to maintaining a high level of MHC II expression on the surface of DCs (Cho et al., 2015).

Recepteur d’origine nantais (RON, also known as MST1R) is a receptor in the tyrosine kinase receptor family of the MET family (Huang et al., 2020). RON participates in epithelial cell proliferation and tissue repair (Nanney et al., 1998), antagonizing LPS-induced inflammatory factor synthesis (Laskin et al., 2010). RON-specific inhibitor BMS777607 is currently undergoing a Phase II clinical trial for cancer research (Khaibullina et al., 2018). There is also evidence that the activation of RON leads to an increase in MHC class II transactivator (CIITA) expression in peritoneal macrophages and regulates the expression of MHC II in macrophages (Wilson et al., 2008).

In this study, we found that RON was expressed in DCs. The inhibition of RON led to increased March-I expression following LPS-mediated DC maturation, enhanced MHC II and CD86 ubiquitination, and inhibited DC maturation.

## Methods

### Mice and Cells

Six-week-old wild-type female C57BL/6 and BALB/C mice were purchased from the Shanghai Laboratory Animal Center (Slaccas, Shanghai, China). The animals were housed in a specific pathogen-free facility at the First Affiliated Hospital of Zhejiang University (Hangzhou, China). The experimental procedures in the use and care of animals were approved by the Ethics Committee of the First Affiliated Hospital of Zhejiang University.

Bone marrow cells were rinsed from the femur and tibia of 6-week-old C57BL/6 mice, and erythrocytes were lysed using a Lysis solution (BD Bioscience, New York, USA, Cat# 559759) and resuspended at 1 × 10^6^ cells/ml in complete RPMI-1640 medium (Gibco, Carlsbad, USA, Cat# 61870044) containing 10% heat inactivated fetal bovine serum (FBS; Gibco, Cat# 10099133), 1% penicillin–streptomycin (Gibco, Cat# 15140122), and 10 mM HEPES (Gibco, Cat# 15630080). The cell suspension was added to an Ultra-Low Attachment Culture Dish (Corning, New York, USA, Cat# 3262) at 20 ml/dish. Finally, 20 ng/ml granulocyte–macrophage colony-stimulating factor (GM-CSF; R&D Systems, Minneapolis, USA, Cat# 415-ML-020), 4 ng/ml interleukin (IL)-4 (R&D Systems, Cat# 404-ML-010), and 5 ng/ml FMS-like tyrosine kinase 3 ligand (FLT3L; R&D Systems, Cat# 427-FL-005) were added to the dish. Splenic DCs were puriﬁed from the splenocytes using a MACS mouse DC isolation kit (Miltenyi Biotech, Bergisch Gladbach, Germany, Cat# 130-108-338). BMS777607 (MCE, Monmouth Junction, USA, Cat# HY-12076) was dissolved in dimethyl sulfoxide (DMSO; Sigma-Aldrich, Darmstadt, Germany, Cat# D2650) to a final concentration of 10 mM and stored at −20°C and was used to inhibit RON activation at a final concentration of 400 nM. Macrophage-stimulating protein (MSP) was purchased from R&D Systems (Cat# 352-MS-010) and used at a finial concentration of 100 ng/ml to activate RON. LPS (100 ng/ml; Sigma-Aldrich, Cat# L2630) was used for DC stimulation. Resident peritoneal macrophages were obtained from C57BL/6 mice by lavaging the peritoneal cavity with 10 ml of RPMI-1640 medium.

### Flow Cytometry and Cell Sorting

For ﬂow cytometry analysis, single-cell suspensions were obtained and incubated with 1 µg/ml Fc block (Biolegend, San Diego, USA, Cat# 101320, RRID: AB_1574975) for 10^6^ cells following staining with the following monoclonal antibodies: BV421-CD11c (clone N418, Cat# 117329, RRID:AB_10897814), APC-CD86 (clone GL-1, Cat# 105011, RRID:AB_493343), PE-MHC II (clone M5/114.5.2, Cat# 107607, RRID:AB_313322). The antibody isotypes were obtained from Biolegend (San Diego, USA). Anti-mouse IgG conjugated with Phycoerythrin (PE) was obtained from Invitrogen (Carlsbad, USA, Cat# A10543, RRID:AB_1500749). The cells were washed three times and stained with fluorochrome-conjugated antibodies at 4°C for 30 min in the dark. An anti-RON antibody (clone E-9, Santa Cruz Biotechnology, Cat# sc-374626, RRID: AB_10989063) was used to detect RON expression in DCs. For intracellular staining, cells were fixed and permeabilized using intracellular staining kits (BD Bioscience) followed by the previously described steps. Data acquisition was performed with a BD Canto II flow cytometer (BD Bioscience, RRID:SCR_012263). Data were analyzed with FlowJo software, version 10.4 (RRID:SCR_008520).

For cell sorting, DCs were incubated with 1 µg/ml Fc block per 10^6^ cells and purified using a MACS mouse DC isolation kit (Miltenyi Biotech). CD3^+^ T cells were puriﬁed from spleen cells using a MACS mouse CD3ϵ^+^ T cell isolation kit (Miltenyi Biotech, Cat# 130-094-973). Cells were isolated quickly and maintained on ice to limit spontaneous DC and CD3*ϵ*^+^ T cell activation.

### Immunoprecipitation and Western Blotting

Cells were lysed with cell lysis buffer (CST, Danvers, USA, Cat# 9803) supplemented with protease and a phosphatase inhibitor cocktail (CST, Cat# 5872). The protein concentration in the extracts was measured using a BCA Protein Assay kit (ThermoFisher Scientific, Waltham, USA, Cat# 23229). Cell lysis, immunoblot analysis, and immunoprecipitation were performed as described previously (Liu et al., 2011). To confirm the expression of RON in DCs, rabbit anti-RON (clone 5029) antibodies were used as the primary antibody in the Western blots as previously described (Wang et al., 2007; Yao et al., 2013a), and GAPDH (Abcam, Cambridge, United Kingdom, Cat# ab181602, RRID:AB_2630358) was used as a loading control. For the MHC II and CD86 ubiquitination assay, bone marrow-derived dendritic cells (BMDCs) were treated with 1 µM MG132 (CST, Cat# 2194), and immunoprecipitated with an MHC II antibody (clone M5/114.5.2, Invitrogen, Cat# 14-5321-82, RRID:AB_467561) or CD86 antibody (clone D-6, Santa Cruz Biotechnology, Cat#sc-28347, RRID:AB_627200), and then immunoblotted with a ubiquitination antibody (clone P4D1, Santa Cruz Biotechnology, Cat# sc-8017, RRID:AB_2762364). At the same time, the lysate was assessed by Western blotting with the following primary antibodies: MHC II (clone M5/114.5.2, Invitrogen) and CD86 (clone D-6, Santa Cruz Biotechnology). The secondary antibodies were: anti-mouse IgG for IP (Abcam, Cat# ab131368), anti-rabbit IgG (Abcam, Cat# ab6721, RRID:AB_955447), and anti-rat IgG (Invitrogen, Cat# A18865, RRID:AB_2535642).

### Quantitative Real-Time-PCR Analysis

Total RNA was extracted from cells using an RNeasy mini column kit (Qiagen, Dusseldorf, Germany, Cat# 74104) according to the manufacturer’s instructions. A sample of 500 ng total RNA was subjected to reverse transcription using 037A (Takara, Dalian, China, Cat# RR037A) according to the instructions. SYBR Green (Bio-Rad, Hercules, USA, Cat# 1725121) was used for q-PCR according to the instructions. Reactions were carried out using a C1000 Thermal Cycler (Bio-Rad), and the cycling parameters were: 15 min at 55°C, 5 min at 95°C, and 40 cycles of 5 s at 95°C and 34 s at 60°C. RT-PCR data were analyzed using a CFX96 Real-Time System. The data were normalized to *GUSB* expression and all RT-PCR experiments were performed in triplicate and repeated independently three times. The primer sequences were: RON forward: 5′-CCTCTGCCGCTGCTTCAAT-3′ and reverse: 5′-GCTGCGTAGGGTATTCGTGG-3′; March-I forward: 5′-AAGAGAGCCCACTCATCACACC-3′ and reverse: 5′-ATCTGGAGCTTTTCCCACTTCC-3′; March-8 forward: 5′-TTCTCAGGATGCCATTTCTGC-3′ and reverse: 5′-GTTGCTTGGATGACTCATGGAA-3′; CIITA forward: 5′-AGGCCTATGCCAACATTGCG-3′ and reverse: 5′-CCATAGCATGCTCTTCCGGG-3′; CD86 forward: 5′-CTGGACTCTACGACTTCACAATG-3′ and reverse: 5′-AGTTGGCGATCACTGACAGTT-3′; GUSB forward: 5′-TTGAGAACTGGTATAAGACGCATCAG-3′ and reverse: 5′-TCTGGTACTCCTCACTGAACATGC-3′.

### Enzyme-Linked Immunosorbent Assay

In ELISA, cells were plated into 24-well plates at a density of 2 × 10^5^ cells/well using serum-free RPMI-1640 medium for 2 h, and the following stimulation was added: LPS + DMSO, LPS + MSP + DMSO, LPS + MSP + BMS777607, and LPS + BMS777607, respectively. The stimulation concentrations were: LPS 100 ng/mL, MSP 100 ng/mL, and BMS777607 400 nM. DMSO was added at the same dose as BMS777607 to control for any effect of DMSO in BMS777607 stimulations. The 24-h cell culture supernatants were analyzed with IL-1β (Dakewe Biotech, Shenzhen, China, Cat# 1210122), IL-12p70 (R&D Systems, Cat#M1270) and IL-10 (R&D Systems, Cat# M1000B) ELISA kits in accordance with the manufacturers’ instructions.

### Mixed Lymphocyte Reaction Assay

BMDCs were treated with LPS + DMSO, LPS + MSP + DMSO, LPS + MSP + BMS777607, or LPS + BMS777607 for 24 h before harvesting, following CD11c magnetic bead sorting, respectively. The stimulation concentrations were: LPS 100 ng/mL, MSP 100 ng/ml, BMS777607 400 nM, and DMSO added at the same dose as BMS777607 to control for any effect of DMSO in BMS777607 stimulations. BMDCs were resuspended in 25 µg/ml mitomycin (Biovision, Palo Alto, USA, Cat# 2713-2) for 20 min. The cells were washed three times with Dulbecco’s phosphate-buffered saline (DPBS; Gibco, Cat# 14190144) and resuspended in complete RPMI-1640 medium containing 2 ng/ml IL-2 (R&D Systems, Cat# 402-ML-020). CD3^+^ T cells were derived from Balb/c mouse splenocytes, sorted with CD3 magnetic beads, resuspended in DPBS, mixed with a final concentration of 2.5 µM carboxyfluorescein succinimidyl ester (CFSE; BD Bioscience, Cat# 565082) for 15 min at 37°C in a water bath, and resuspended in complete RPMI-1640 medium containing 2 ng/ml IL-2. A density of 3 × 10^5^ CD3^+^ T cells/well was added to a U-shaped 96-well plate. BMDCs were added at a ratio of 1:10 or 1:50 (DC:T cells). T cells stained with CFSE were used as a negative control. After co-culturing for 48 h, the cells were collected and data acquisition was performed with a BD Canto II flow cytometer. Data were analyzed with FlowJo software, version 10.4.

### Statistical Analysis

SPSS software was used to perform regular statistical analysis. A one-way analysis of variance with a Games–Howell’s multiple comparison test was used for multiple comparisons. The data represent the mean ± SEM of three independent experiments. *p < 0.05; **p < 0.01; ***p < 0.001.

## Results

### RON Expression in DCs

**Figure 1:**
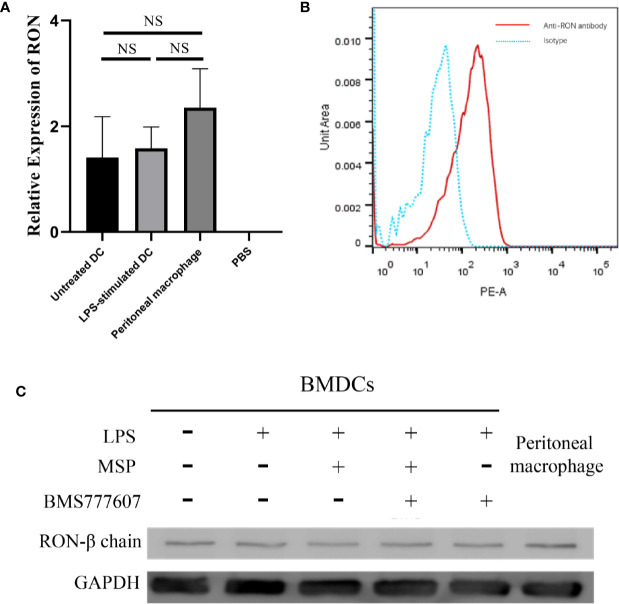
The Recepteur d’origine nantais expression in DCs. **(A)** RT-PCR analysis of RON mRNA expression from untreated DCs, LPS-stimulated DCs, and peritoneal macrophages. GUSB expression was used as an internal control and normalized to relative RON expression, and the data represent the mean ± SEM of three independent experiments. NS, no significant difference. **(B)** DCs were purified with a MACS mouse DC isolation kit and cultured for 2 h in RPMI-1640 medium without FBS. The total protein in the cell lysates was assessed by Western blotting with the specific anti-RON antibody, 5029. Peritoneal macrophages were used as a positive control to confirm that RON was expressed in DCs. **(C)** DCs were incubated with 1 µg/ml Fc block per 10^6^ cells and purified by a MACS mouse DC isolation kit as described in the *Methods*. The cells were fixed and permeabilized using intracellular staining kits, and then incubated with an anti-RON antibody (clone E-9) or mouse IgG as an isotype, following incubation with anti-mouse IgG conjugated with PE. Cells were harvested and data acquisition was performed with a BD Canto II flow cytometer. Data were analyzed with FlowJo software, version 10.4. The experiments were repeated twice independently to confirm the results.

To assess whether RON was expressed in DCs, we examined the level of RON mRNA expression by RT-PCR, which demonstrated that RON was expressed in both untreated DCs and LPS-stimulated DCs (**Figure 1A**). The transcriptional levels of RON in DCs were similar to those in peritoneal macrophages and were not affected by LPS stimulation (**Figure 1A**). Western blotting results showed that RON was expressed in DCs and RON expression was not influenced by LPS, MSP, or BMS777607 treatment (**Figure 1B**). Flow cytometry assay results also showed RON expression in DCs using the mouse anti-RON antibody, E-9 (**Figure 1C**).

### RON Inhibition Downregulates MHC II and CD86 Expression in BMDCs and Splenic DCs, Which Is Inconsistent With the Transcription Results for MHC II and CD86

**Figure 2:**
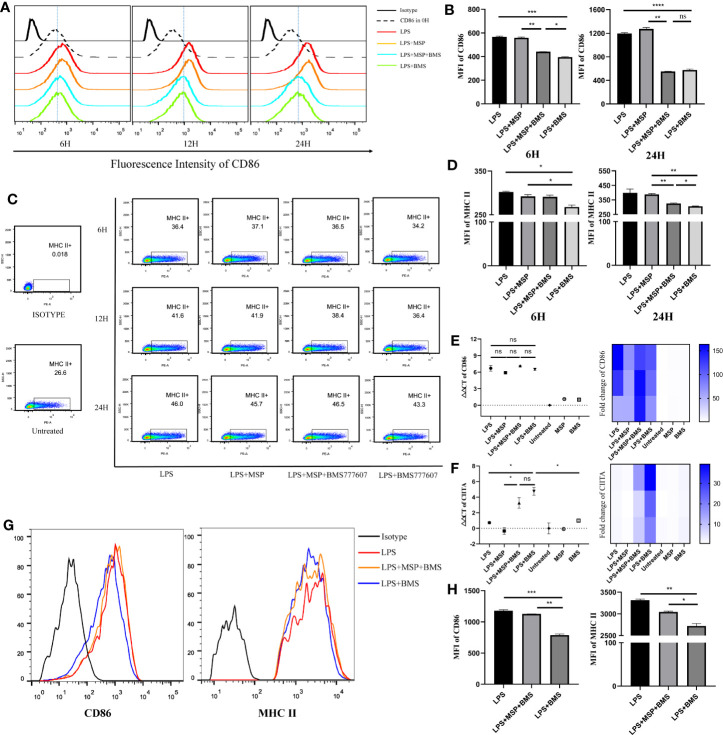
RON inhibition downregulates MHC II and CD86 in BMDCs and splenic dendritic cells, which is inconsistent with transcription results for MHC II and CD86. **(A−D)** On day 9, bone marrow-derived DCs were treated for 6, 12, and 24 h before harvesting, respectively. **(A)** Representative histogram of flow cytometry analysis of CD86 expression in BMDCs for CD11c^+^ gated cells. **(B)** Mean fluorescence intensity (MFI) of CD86 on BMDCs from **(A)**. **(C)** The expression of MHC II molecules was determined by flow cytometry for CD11c^+^ gated cells and the value of MHC II shown in the scatter plot represents the percentage of cells. **(D)** MFI of MHC II on BMDCs from **(C)**. **(E, F)** DCs were harvested and cultured for 24 h in complete RPMI-1640 medium with stimulation, respectively. The total mRNA was collected with an RNeasy mini column kit and the level of CD86 and CIITA mRNA was tested by RT-PCR. GUSB expression was used as an internal control. Heat map of gene expression for CD86 and CIITA, comparing unstimulated DCs are shown. **(G, H)** Splenic DCs were isolated from spleen cells of C57/B6 mice and treated with LPS, LPS + MSP + BMS, or LPS + BMS for 24 h, respectively. **(G)** Representative histogram of flow cytometry analysis of MHC II and CD86 expression in splenic DCs. **(H)** MFI of CD86 and MHC II from **(G)**. The data represent the mean ± SEM of three independent experiments. *p < 0.05; **p < 0.01; ***p < 0.001; ****p < 0.0001; NS, no significant difference.

We then detected changes in DC surface marker expression by flow cytometry following stimulation with LPS 100 ng/ml, MSP 100 ng/ml, and BMS777607 400 nM. The level of CD86 and MHC II expression was increased in each group stimulated with LPS compared with untreated DCs. The results of the MFI assay suggested that both CD86 (**Figures 2A, B**) and MHC II (**Figures 2C, D**) expression decreased with RON inhibition after stimulation for 12 or 24 h. We examined changes in the level of CD86 and CIITA mRNA expression. CD86 was not significantly altered following RON inhibition (**Figure 2E**). CIITA expression was increased by almost 13 times when 400 nM BMS777607 was used to inhibit RON (p < 0.05) compared with LPS-treated DCs (**Figure 2F**). We found that RON inhibition leads to decreased MHC II and CD86 in LPS-mediated splenic DCs after stimulation for 24 h (**Figures 2G, H**). The inhibition of RON downregulated the expression of MHC II (p < 0.01) and CD86 (p < 0.001) in LPS-mediated splenic DCs, and these effects were antagonized by MSP.

### The Inhibition of RON Enhances CD86 and MHC II Ubiquitination

**Figure 3:**
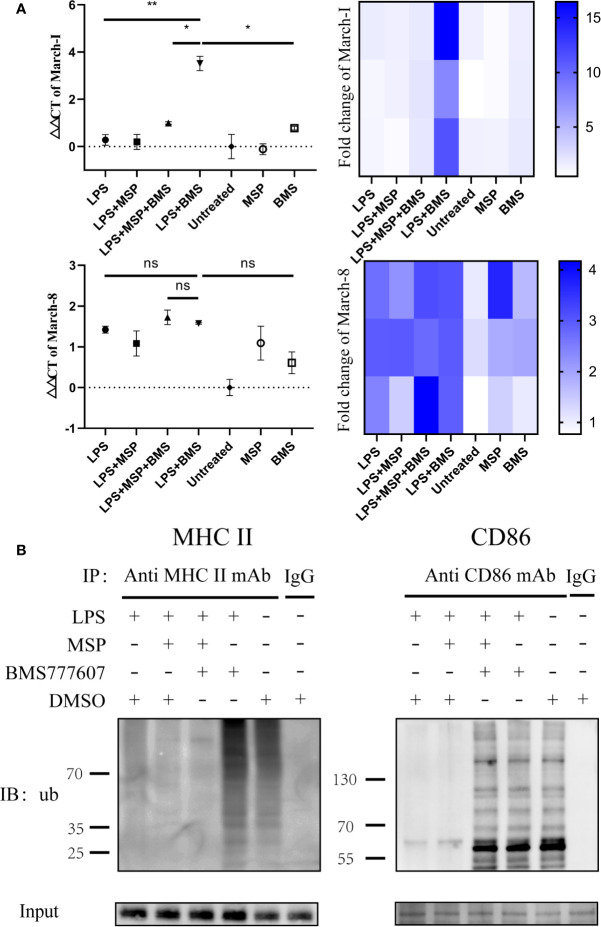
Inhibition of RON leads to increased transcription of March-I without affecting March-8, resulting in increased MHC II and CD86 ubiquitination. **(A)** DCs were stimulated for 4 h and total mRNA was collected using an RNeasy mini column kit. The level of March-I and March-8 mRNA was tested by RT-PCR and GUSB expression was used as an internal control. The data represent the mean ± SEM of three independent experiments. NS, No significant difference; *p < 0.05; **p < 0.01. Heat map of gene expression for March-1 and March-8, comparing unstimulated DCs are shown. **(B)** DCs were stimulated for 4 h and 1 µM MG132 was added at the same time. The immune precipitates were analyzed by immunoblotting using anti-ubiquitin antibodies and the secondary antibody was anti-mouse IgG for immunoprecipitation to eliminate the effects of light and heavy chains. The experiment was repeated twice independently.

Since CD86 and MHC II transcription and protein level data were inconsistent, we next considered whether the degradation of these two molecules had increased. MHC II and CD86 are ubiquitinated by the E3 ligases, March-I and March-8 (Oh and Shin, 2015). Thus, we sought to determine the changes in the gene expression of March-I and March-8. RON inhibition increased March-I expression following treatment with LPS (p < 0.01); this inhibitory effect was antagonized by MSP (p < 0.05) (**Figure 3A**), whereas March-8 did not exhibit significant changes (**Figure 3A**). In addition, this phenomenon was not observed in the non-LPS-treated group. Therefore, we performed an immunoprecipitation assay of CD86 and MHC II and examined the changes in the level of ubiquitination of these two molecules. MHCII and CD86 could be polyubiquitinated with 7 kDa ubiquitin (Lei et al., 2018), which resulted in proteins of various molecular weights appearing in the lane. Similar to the RT-PCR results, the ubiquitination of these two molecules increased with RON inhibition and MSP stimulation reduced the degree of ubiquitination induced by BMS777607 (**Figure 3B**).

### RON Inhibition Reduced the Release of IL-1β and IL-12p70, as Well as Increased the Release of IL-10 in LPS-Mediated DCs and an Allogeneic Mixed Lymphocyte Reaction Assay Revealed Reduced BMDC Maturity

**Figure 4:**
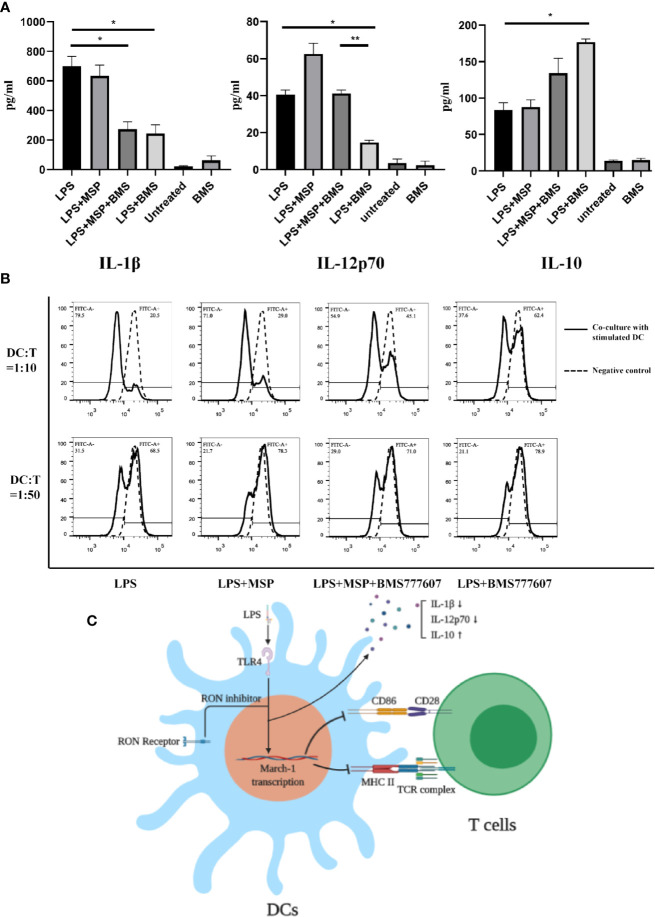
**(A)** IL-1β, IL-12p70, and IL-10 production by LPS-treated BMDCs in the presence of a RON inhibitor leads to downregulation of the ability of BMDCs stimulated with LPS to proliferate T cells. BMDCs were cultured in serum-free RPMI-1640 medium for 2 h and then stimulated with LPS, LPS + MSP, LPS + MSP + BMS777607, LPS + BMS777607, BMS777607 or unstimulated respectively. The supernatant was collected at 24 h and the assay was performed as described in the *Methods*. The data represent the mean ± SEM of three independent experiments. NS, no significant difference; *p < 0.05; **p < 0.01. **(B)** The allogeneic MLR protocol was performed as described in the *Methods* section. The left peak indicates a T cell after proliferation and division. The negative control was CFSE-stained T cells without co-culture with DCs. The experiment was repeated twice independently. **(C)** Scheme showing that the use of RON inhibitors leads to an increased March-I transcription and thus an increased CD86 and MHC II ubiquitination when LPS stimulated DCs. These changes led to a decrease of IL-1β and IL-12p70 release and increased IL-10 release. Moreover, the ability of LPS-stimulated DCs to stimulate T cell proliferation was decreased when the inhibition of RON.

To evaluate the function of DCs after RON inhibition, we next examined the level of cytokine release from DCs following LPS stimulation. RON inhibition significantly reduced the level of IL-1β (p < 0.05) and IL-12p70 (p < 0.05) and increased the level of IL-10 (p < 0.05) in the cell culture supernatants of LPS-treated BMDCs after 24 h (**Figure 4A**). Treatment with MSP antagonized the reduction of IL-12p70 release (p < 0.01) after RON inhibition in DCs (**Figure 4A**).

Finally, we performed co-culture experiments with BMDCs and splenic T cells isolated from Balb/c mice. The results showed that the ability of BMDCs to stimulate allogeneic T lymphocytes was significantly reduced following RON inhibition (**Figure 4B**), and MSP was used to antagonize this process. Similar results were obtained when we used a DC:T cell ratio of 1:10 or 1:50 for co-cultivation and the experiment was repeated twice, independently.

## Discussion

DCs are the only immune cell that can activate naive T cells and they play an important role in various tissues and organs. Untreated DCs express low levels of CD86 and MHC II but are able to capture antigens via endocytosis and the Fc receptor (Pearce and Everts, 2015). After processing, the antigen fragment is loaded into the MHC II molecule and expressed on the cell surface. At the same time, when receiving pathogen-associated molecular pattern stimulation, DCs show increased surface expression of MHC II and CD86, providing the first and co-stimulatory signals required for T cell activation, resulting in the activation of naive T cells. LPS-stimulated DCs also secrete IL-12p70, Il-1β, and other cytokines to assist in the activation of other immune cells (Liu and Cao, 2016).

RON is expressed on peritoneal macrophages (Yao et al., 2013b), osteoclasts (Andrade et al., 2017), and Kupffer cells (Stuart et al., 2011). Here, we determined that RON is expressed on both DCs and LPS-stimulated DCs, and was not influenced by LPS (**Figures 1A, B**). Interestingly, when the MHC II and CD86 phenotypes were detected by flow cytometry, we found that RON-inhibited DCs exhibited lower levels of MHC II and CD86 when treated with LPS in both BMDCs and splenic DCs (**Figures 2A–D, G, H**). These flow cytometry results did not match the above CIITA and CD86 transcription results (**Figures 2E, F**). Based on this contradiction, we suspected that RON inhibition can lead to the ubiquitination and degradation of both MHC II and CD86 by the E3 ligases, March-I and/or March-8. Our RT-PCR results showed that the inhibition of RON resulted in increased transcription of the E3 ligase March-I, whereas March-8 transcription remained unchanged (**Figure 3A**). Subsequent Western blotting results confirmed that there was an increase in the degree of MHC II and CD86 ubiquitination when RON was inhibited in DCs (**Figure 3B**). These findings provide evidence that although CIITA transcription increases with RON inhibition, the ubiquitination of MHC II and CD86 increases, leading to the degradation of these two molecules and ultimately decreased levels of MHC II and CD86 (**Figure 4C**).

Mitogen-activated protein kinase (MAPK) inhibition leads to increased CIITA transcription (Yao et al., 2006) and RON inhibition may lead to a decrease in extracellular signal-related kinase (Erk)1/2 phosphorylation and an increase in CIITA transcription, as described previously (Wilson et al., 2008). Interestingly, when the MHC II and CD86 phenotypes were detected by flow cytometry, we found that RON-inhibited DCs exhibited lower levels of MHC II and CD86 than the LPS-stimulated DCs (**Figures 2A–H)**. These flow cytometry results did not match the above CIITA and CD86 transcription results (**Figures 2E–F**). We suspected that RON inhibition leads to the ubiquitination and degradation of both MHC II and CD86 by March-I and/or March-8, which would suggest that E3 ligases can ubiquitinate MHC II and CD86 in DCs. Our RT-PCR results show that the inhibition of RON resulted in increased transcription of the E3 ligase March-I, whereas March-8 transcription remained unchanged (**Figure 3A**). Western blotting results confirmed that there was an increase in the degree of MHC II and CD86 ubiquitination on inhibiting RON in DCs (**Figure 3B**). To our surprise, we only observed CD86 ubiquitination but not MHC II ubiquitination in the LPS + MSP + BMS777607 group (**Figure 3B**), in which the transcription of March-I did not increase significantly (**Figure 3A**).

Therefore, these findings suggest that RON maintains LPS-stimulated DC activation by inhibiting March-I expression in LPS-stimulated DCs. Since the release of IL-1β and IL-12p70 is a marker of DC maturation, we measured the concentrations of IL-1β and IL-12p70 released by LPS-stimulated DCs. The ability of RON-inhibited LPS-stimulated DCs to release IL-1β and IL-12p70 was significantly reduced compared with normal LPS-stimulated DCs (**Figure 4A**). In addition, we evaluated IL-10 production, and the results showed increased release of IL-10 in LPS-stimulated DCs when RON was inhibited (**Figure 4A**). At the same time, we performed an allogeneic mixed lymphocyte reaction experiment, and the results showed that RON-inhibited DC stimulation of T cell proliferation was significantly decreased (**Figure 4B**).

Our findings may provide novel insight into the role of RON receptors in both innate and acquired immunity. The RON receptor may protect the process of DC differentiation when receiving external stimuli. As a tyrosine kinase receptor, RON has an important protective effect on HK2 cells from apoptosis caused by H_2_O_2_ (Lee et al., 2013a; Lee et al., 2013b), and protected peritoneal macrophages from LPS-induced apoptosis (Chen et al., 2002). RON inhibition enhances LPS-induced apoptosis in DCs, which may contribute to the increased March-I transcription. However, we have been unable to clarify how RON promotes March-I transcription. This research has been impeded by the lack of a RON knockout mouse and we could only use BMS777607 to inhibit RON activation, which may have an impact on the reliability of the experimental results. Thus, further evidence is required.

In the present study, we provide evidence that RON is expressed in DCs. Our findings show that a blockade in RON signaling can lead to enhanced March-I transcription. This results in increased MHC II and CD86 ubiquitination and decreased DC maturity.

## Data Availability Statement

All datasets generated for this study are included in the article and/or the supplementary material.

## Ethics Statement

The experimental procedures regarding the use and care of animals were approved by the Ethics Committee of the First Affiliated Hospital of Zhejiang University.

## Author Contributions

LH, XZ, and HY performed DC isolation and analysis, ELISA, RT-PCR, flow cytometry, and Western blotting. HY, QF, XF, and WW supervised the study, analyzed experimental data, prepared figures and tables, and wrote the manuscript. All authors approved the manuscript for submission and publication.

## Funding

The work was supported by the National Natural Science Foundation of China grant #81872883 (to HY), National Science & Technology Major Project of China grant #2017ZX10204401 (to QF), Zhejiang Provincial Natural Science Foundation of China grant #LQ19H190001 (to XZ), and Medical Health Science and Technology Project of Zhejiang Province Health Commission grant #2019KY381 (to WW). The funders had no role in the study design, data collection and analysis, decision to publish, or preparation of the manuscript.

## Conflict of Interest

The authors declare that the research was conducted in the absence of any commercial or financial relationships that could be construed as a potential conflict of interest.
